# Osteoclast inhibitors to prevent bone metastases in men with high-risk, non-metastatic prostate cancer: A systematic review and meta-analysis

**DOI:** 10.1371/journal.pone.0191455

**Published:** 2018-01-25

**Authors:** Aimee R. Hayes, Daniel Brungs, Nick Pavlakis

**Affiliations:** 1 Department of Medical Oncology, Royal North Shore Hospital, St Leonards, New South Wales, Australia; 2 Department of Medical Oncology, Illawarra Cancer Care Centre, Wollongong Hospital, Wollongong, New South Wales, Australia; 3 Illawarra Health and Medical Research Institute, University of Wollongong, Wollongong, New South Wales, Australia; 4 Sydney Medical School, University of Sydney, Sydney, New South Wales, Australia; University of L'Aquila, ITALY

## Abstract

**Background:**

In advanced prostate cancer, osteoclast inhibitors prevent and palliate skeletal related events associated with bone metastases. However, it is uncertain whether they play a disease-modifying role earlier in the course of the disease.

**Methods:**

Medline, EMBASE, Cochrane Central Register of Controlled Trials and Cochrane Database of Systematic Reviews and ASCO conference proceedings were searched for randomized controlled trials that compared osteoclast inhibitors with placebo and/or standard of care (SOC) in patients with high-risk, non-metastatic prostate cancer. The primary outcome measure was incidence of new bone metastases; secondary outcomes included overall survival (OS), prostate cancer specific survival, mortality unrelated to prostate cancer, toxicity and health related quality of life outcomes. Results are presented as relative risk (RR) with 95% confidence intervals (CI).

**Results:**

Six randomized controlled trials (5947 participants) were included, five evaluating bisphosphonates and one denosumab. Overall, there was no difference in incidence of bone metastases between participants treated with osteoclast inhibitors versus placebo/SOC (RR 1.09, 95%CI 0.84–1.41, p = 0.51) however significant heterogeneity was observed between studies. The denosumab trial was the largest and only positive trial amongst the included studies (RR 0.83, 95%CI 0.73–0.95, p = 0.007). No significant difference was observed in OS (RR 0.99 95% CI 0.89–1.10, p = 0.84) nor prostate cancer specific survival (RR 1.12 95%CI 0.93–1.36, p = 0.24). Most studies reported increased rates of osteonecrosis of the jaw (5% or less) and hypocalcemia (2% or less) with osteoclast inhibitors.

**Conclusions:**

While there is limited evidence that bisphosphonates alter the natural history of high-risk, non-metastatic prostate cancer, denosumab delays onset of bone metastases in this patient population. Neither class of osteoclast inhibitor demonstrated an impact on survival outcomes. Future trials with better defined patient selection and a robust definition for high risk disease is critical.

## Introduction

Prostate cancer is the second most common cancer in men worldwide [1] and, in advanced disease, the skeleton is the dominant site of metastases [2]. Bone metastases can cause significant morbidity including pain, pathological fractures, spinal cord compression and occasionally, hypercalcemia. In addition, hospitalization with skeletal related events (SREs) is associated with high health economic burden [3].

A cornerstone of management in men with advanced disease includes the prevention and palliation of SREs. The nitrogen-containing, third generation bisphosphonate, zoledronic acid, became standard of care for men with castrate resistant prostate cancer (CRPC) and bone metastases after a phase III trial demonstrated a 35% reduction in SREs compared with placebo; a composite endpoint that included pathologic fractures, radiotherapy to bone, surgery to bone, and spinal cord compression [4]. In a similar population, denosumab, a receptor activator of nuclear factor kappa-B (RANK) ligand antibody, was later compared to zoledronic acid and found to further prolong the time to first SRE (20.7 months versus 17.1 months; p = 0.008 for superiority) [5].

Recently, there has been increasing interest in the role of osteoclast inhibitors in the prevention of bone metastases. The bone dominant nature of prostate cancer is thought to arise from the complex interactions between tumor cells and the bone microenvironment [6, 7]. Activation of osteoclasts has been shown to play an important role in the development of prostate cancer bone metastases [8, 9]. Furthermore, preclinical studies suggest that the inhibition of osteoclast activation may impede the development or progression of bone metastases [10–12]. A phase III trial published by Smith *et al* [13] was the first clinical study to demonstrate a significant delay in the development of bone metastases with denosumab compared with placebo in patients with CRPC (33.2 versus 29.5 months; HR 0.84, p = 0.032).

Prevention of bone metastases remains a major unmet clinical need. Following the introduction of prostate specific antigen (PSA) screening, the rates of men presenting with metastatic disease is falling and the opportunity to intervene and alter the natural history of prostate cancer in select, high-risk patients has never been greater. The role of osteoclast inhibitors in the prevention of bone metastases in men with high-risk, non-metastatic (M0) prostate cancer remains uncertain. We aimed to assess the effects of osteoclast inhibitors on incidence of new bone metastases and the relative harms of treatment in this population.

## Methods

### Study protocol and registration

This study has no protocol. Methods are reported according to the Preferred Reporting for Systematic Reviews and Meta-Analyses (PRISMA) guidelines (S1 PRISMA Checklist) [14].

### Study selection criteria

We included all randomized controlled studies that compared bisphosphonates or denosumab with placebo and/or standard of care (SOC) in patients with high-risk, M0 prostate cancer. For inclusion in the quantitative analysis, studies were required to compare the efficacy of osteoclast inhibitors on incidence of bone metastases or overall survival. The primary outcome measure was incidence of bone metastases; secondary outcomes included overall survival, prostate cancer specific survival, mortality unrelated to prostate cancer, toxicity and health related quality of life (HRQOL) outcomes.

### Search strategy

The following databases were searched for randomized controlled trials fulfilling the above criteria: Medline (1946 to October 13, 2016), EMBASE (1974 to October 13, 2016), Cochrane Central Register of Controlled Trials and Cochrane Database of Systematic Reviews and ASCO conference proceedings. The search strategy can be found in S1 Appendix and was designed to maximize sensitivity. Bibliography searches of identified studies were then checked for additional source material. Searches were restricted to English language publications.

### Quality and risk of bias assessment

Each study was assessed for quality and bias using the Cochrane Collaboration Risk of Bias Tool [15] and the PRISMA guidelines [14], with particular focus on the domains of random sequence generation, allocation concealment, allocation and outcome blinding, withdrawals and loss to follow-up, intention-to-treat analysis and selective reporting. The results were presented as risk of bias tables, a risk of bias summary and risk of bias graph.

### Data collection

Two authors (AH, DB) independently reviewed full-text manuscripts of included studies and extracted data related to study characteristics and study quality with disagreement resolved by consensus with a third author (NP). The specific documented details included accrual period, median follow-up, country of study, sample size, methods of randomization, blinding, withdrawals, completeness of follow-up, use of intention-to-treat analysis, included definitions of high-risk prostate cancer, mean age, castration sensitive or refractory disease status, Gleason score, disease stage, baseline PSA, prior local therapy, osteoclast inhibitor regimen and compliance, methods of evaluation of bone metastases and follow-up, primary and secondary endpoints and outcomes.

### Summary measures and methods of analysis

The principal summary measure used for the meta-analyses was risk ratio using the Mantel-Haenszel method and a random effect model using RevMan 5.3 analysis software (The Nordic Cochrane Centre, The Cochrane Collaboration, Copenhagen, Denmark). Point estimate of risk ratios and 95% confidence intervals (CI) are provided along with forest plots. Statistical heterogeneity was assessed using the I^2^ statistic. Formal analysis of publication bias was not performed due to insufficient number of trials in any comparison. Pre-specified subgroup analysis for incidence of bone metastases was performed based on drug class of osteoclast inhibitor given their different mechanisms of action.

## Results

### Study selection

**Fig 1:**
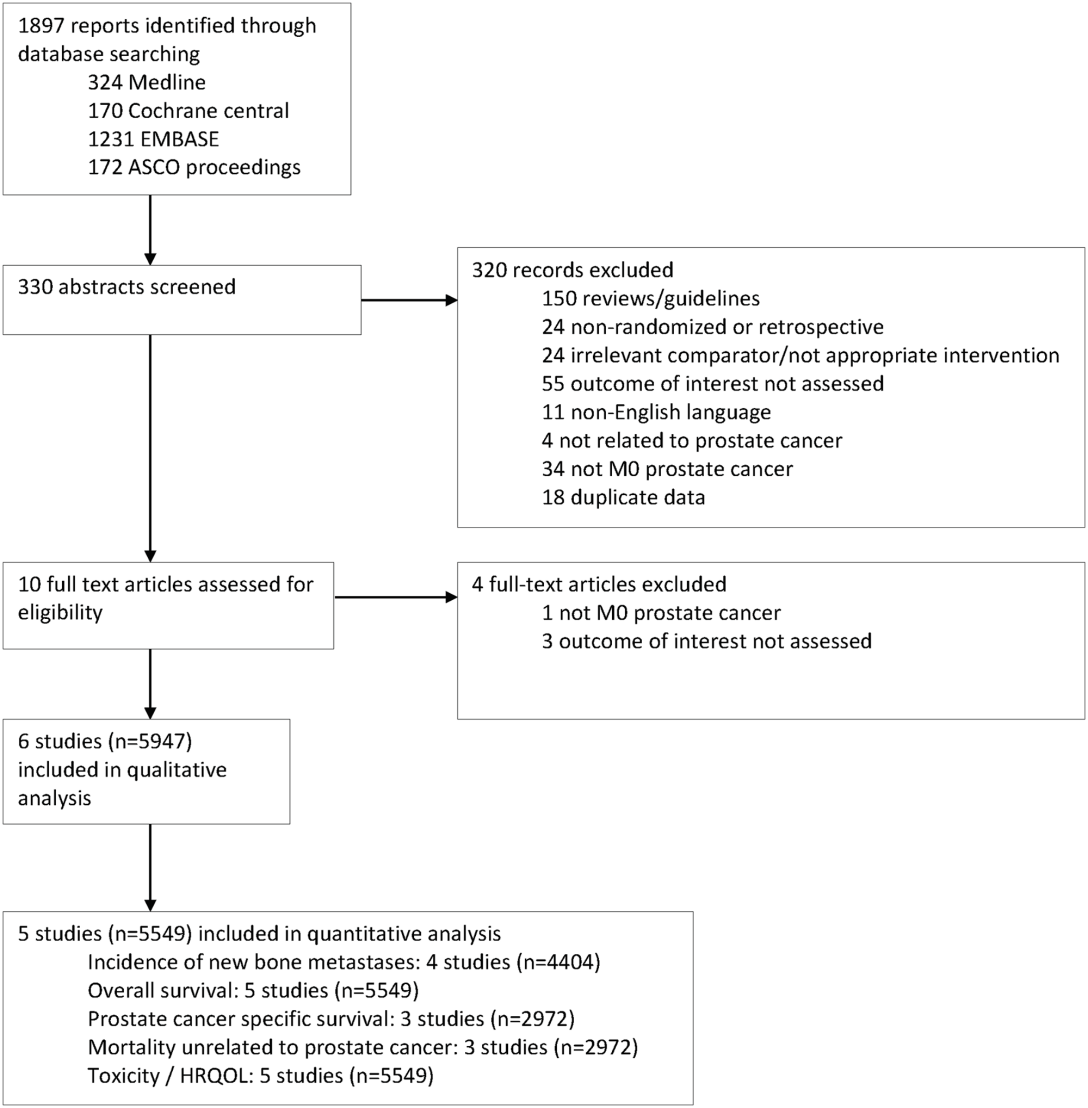
Flow diagram of study selection.

The search criteria identified 1,897 potential studies for inclusion, with 330 studies selected for abstract review, and 10 selected for full-text review (Fig 1). The majority of studies were not suitable for inclusion as they were not randomized trials, had an irrelevant comparator or inappropriate outcome, did not include participants with non-metastatic prostate cancer or were duplicate data. Of the 10 studies included for full-text review, three did not study an appropriate outcome and one did not include participants with M0 prostate cancer.

### Study characteristics

Six studies (5,947 participants) met the inclusion criteria of the systematic review and were included for quality assessment and data extraction (Fig 1). One study (398 participants) was terminated early due to low event rate and did not have published data suitable for inclusion in the pooled analysis [16, 17].

The range of median follow-up was 3.6–11.5 years (Table 1). The study sample sizes ranged from 398–1432 participants. The age (median 65–74 years) and performance status (ECOG 0/1 96–100%) of participants was similar between the trials but the patient characteristics denoting risk of bone metastases significantly varied (Table 2). There were also differences in mechanism of action of osteoclast inhibitor, frequency of drug administration and differences in standard of care regimens across the studies (Table 1 and S1 Table).

**Table 1:** Characteristics of included studies.

Trial	Median follow-up (years)	*n*	Treatment arm	Control arm	Primary outcome
**Zometa 704** [16, 17]	Trial terminated after 3 years	398*	ZA 4 mg q4weekly for 49 cycles	Placebo	Time to first bone metastasis
**MRC PR04** [18, 19]	11.5	508	Clodronate 2080 mg/day for up to 5 years	SOC (Radiotherapy and/or ADT) + Placebo	Time to first symptomatic bone metastasis or prostate cancer death
**Smith 2012** [13]	NR	1432	Denosumab 120 mg q4weekly	SOC (ADT +/- antineoplastic agents) + Placebo	Time to first bone metastasis (symptomatic or asymptomatic) or death from any cause
**TROG 03.04 RADAR** [20]	7.4	1071	ZA 4 mg q3monthly for 18 months	Short (6 mo)- or intermediate (18 mo)-term ADT + definitive radiotherapy	Prostate cancer specific mortality
**ZEUS** [21]	4.8	1393	ZA 4 mg q3monthly for up to 4 years	SOC (ADT)	Incidence of bone metastases on bone imaging at 4 ± 0.5 years
**STAMPEDE** [22]	3.6	1145 (M0); 1817 (M1)	ZA (4 mg every 3 weeks for 6 cycles, then q4weekly until 2 years); docetaxel^†^ and ZA	SOC (ADT); Radiotherapy at 6–9 mo post-randomization^‡^	Overall survival and failure free survival

Abbreviations: M0, non-metastatic; M1, distant metastatic disease; ADT, androgen deprivation therapy; ZA, zoledronic acid; SOC, standard of care; NR, not reported

* Planned accrual 991

^†^ 75 mg/m^2^ q3weekly for 6 cycles with prednisone 10 mg daily

^‡^ Radiotherapy at 6–9 months after randomization was encouraged for participants with N0M0 disease until Nov 2011, then mandated. Radiotherapy was optional for participants with N+M0 disease.

**Table 2:** Participant characteristics denoting metastasis risk.

Trial	Stage and high risk definition	Hormone status	Gleason score > 7	Median PSA at randomization (μg/L)	N+ disease	T3/4 disease	Prostatectomy and/or radiotherapy
**Zometa 704** [16, 17]	M0; rising PSA despite ADT	Castrate resistant	29%	13.8	17%	NR	34%
**MRC PR04** [18, 19]	M0; T2–4	Castrate sensitive	NR	13.0 (TG); 10.0 (CG)	3%	47%	71%
**Smith 2012** [13]	M0; rising PSA despite ADT; PSA ≥8 μg/L and/or PSA DT ≤10 mo	Castrate resistant	32%	12.2	13%	NR	45%
**TROG 03.04 RADAR** [20]	M0; T2a (Gleason >7 and PSA ≥10 μg/L); or T2b–4, N0	Castrate sensitive	35%	14.0–15.0*	0%^‡^	37%	Radiotherapy planned in 98%
**ZEUS** [21]	M0; Gleason 8–10 or pN+ or PSA at diagnosis >20 μg/L	Castrate sensitive	62%	40.0^†^	24%	NR	55%
**STAMPEDE** [22]	M1 or M0; high-risk (at least two of T3/4, Gleason 8–10 or PSA ≥40 μg/L) starting ADT	Castrate sensitive	71%	65.0	50%	82%	60%^§^

Abbreviations: M0, non-metastatic; M1, distant metastatic disease; PSA, prostate specific antigen; DT, doubling time; ADT, androgen deprivation therapy; TG, treatment group; CG, control group; SOC, standard of care; NR, not reported

* 14.9 (Short term androgen suppression control group), 14.0 (Short term androgen suppression treatment group), 15.0 (Intermediate term androgen suppression control group), 14.0 (Intermediate term androgen suppression treatment group)

^†^ Mean PSA at diagnosis

^‡^ N+ disease excluded

^§^ Radiotherapy reported in 60% of M0 cohort

For the MRC PR04 trial [19], data for overall survival was taken from the initial study (median follow-up 118 months) as published data suitable for calculation of risk ratio was not available from the follow-up study (median follow-up 138 months) [18]. STAMPEDE [22] included patients with M0 and M1 disease, however, only those participants with M0 disease were included in the quantitative analysis. STAMPEDE [22] incorporated incidence of bone metastases into a composite outcome, failure free survival, and therefore could not be included in the primary outcome quantitative analysis.

#### Treatment arm

Five studies included a treatment drug regimen with a bisphosphonate; MRC PR04 [19] used the first generation, non-nitrogenous oral bisphosphonate, clodronate, while the other four studies [16, 20–22] used the more potent, third generation, nitrogen-containing bisphosphonate, zoledronic acid administered intravenously. The frequency of zoledronic acid administration varied between 4 mg every 4 weeks and every 12 weeks. Treatment duration varied between 18 months and 4 years. One study [13] investigated denosumab, which binds and inhibits RANK ligand, thus inhibiting RANK pathway signaling, which in turn inhibits osteoclast differentiation and activation.

#### Control arm

The control regimen in all studies was SOC and/or placebo. There was variation between the studies in terms of what treatments constituted standard of care (Table 1 and S1 Table). For example, STAMPEDE [22] and RADAR [20] included ADT and radiotherapy as SOC. The Zometa 704 [16] and Smith 2012 [13] trials allowed prior or concomitant therapy deemed necessary by the treating clinician including antineoplastic agents.

#### Definition of high-risk prostate cancer

There was large heterogeneity in definitions of high-risk prostate cancer across the studies. The Zometa 704 [16, 17] and Smith 2012 [13] trials, both required PSA to be rising despite ADT and hence were studies of castrate resistant disease, in contrast to the other studies which included participants with castrate sensitive disease. Smith 2012 [13] also specified that PSA must be greater than 8 μg/L and/or have a doubling time ≤10 months denoting particularly high risk. The ZEUS [21], RADAR [20] and STAMPEDE [22] trials used different combinations of tumor stage, Gleason score and PSA score to define high risk but PSA doubling time was not specified as an inclusion criteria. The MRC PR04 [19] only included tumor stage as part of the high risk definition. Hence, there was large variation between the studies in proportion of participants with Gleason score >7, N+ disease status and median PSA at baseline (Table 2). Rates of prior prostatectomy and/or radiotherapy also varied between the trials (Table 2).

### Risk of bias

The overall quality was good for the trials included in the quantitative analysis (S2 Table, S1 and S2 Figs). The Zometa 704 [16, 17] was difficult to assess due to paucity of published data; there was no information provided on random sequence generation, allocation concealment, blinding of outcome assessment, incomplete outcome data and selective reporting. The STAMPEDE [22], RADAR [20] and ZEUS [21] trials were open-labeled and not placebo-controlled and hence were at risk of performance bias. Smith 2012 [13], ZEUS [21] and Zometa 704 [16] had good descriptions of the imaging schedule and what clinical parameters or symptoms would prompt repeat imaging whereas STAMPEDE [22], RADAR [20] and MRC PR04 [19] did not.

For all the studies, except Smith 2012 [13] and ZEUS [21], it was unclear if there was blinding of outcome assessment in regards to new bone metastases. Only Smith 2012 [13] reported central radiology review in the entire study cohort. ZEUS reported central radiology review in a subset of patients. In addition, only Smith 2012 [13], ZEUS [21] and Zometa 704 [16] reported that bone metastases seen on bone scan were confirmed on plain radiographs, computed tomography (CT) or magnetic resonance imaging (MRI). The MRC PR04 [19] did not report how symptomatic bone metastases were confirmed radiologically.

All studies had acceptable losses to follow-up and all studies, except Zometa 704 [16, 17], reported intention-to-treat analysis. There were no studies at risk of selective reporting bias.

### Measurement of effect

#### Incidence of new bone metastases

**Fig 2:**
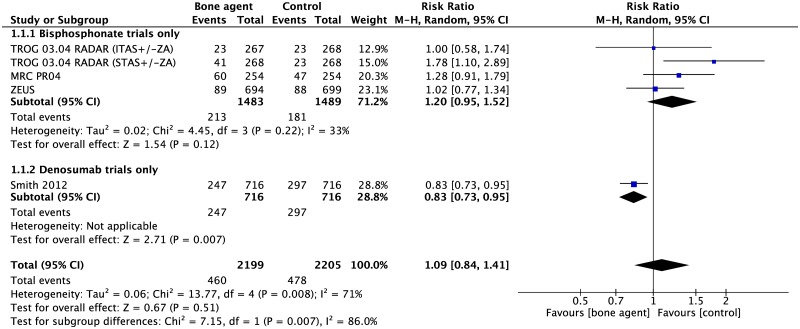
Forest plot for incidence of new bone metastases. Abbreviations: ITAS, intermediate term androgen suppression; STAS, short term androgen suppression; ZA, zoledronic acid.

Four studies (4,404 participants) investigated the effect of osteoclast inhibitors on incidence of new bone metastases. There was no evidence of a difference in incidence of new bone metastases between osteoclast inhibitors and placebo and/or SOC when all four studies were pooled (RR 1.09, 95%CI 0.84–1.41, p = 0.51). However, this method resulted in substantial heterogeneity (I^2^ 71%, p = 0.008) between studies (Fig 2).

A subgroup analysis of only the bisphosphonate trials showed reduced heterogeneity (I^2^ 33%) however there was no difference in incidence of bone metastases between bisphosphonates and placebo/SOC (RR 1.20, 95%CI 0.95–1.52, p = 0.12). The STAS (short term androgen suppression) arm of the RADAR trial is noted to be an outlier and when this is removed from the analysis, the heterogeneity is reduced (RR 1.10, 95%CI 0.90–1.34, p = 0.35, I^2^ 0%) (S3 Fig).

The denosumab trial [13] is the largest and only positive trial amongst the included studies (RR 0.83, 95%CI 0.73–0.95, p = 0.007) and denosumab has a significantly different mechanism of action compared with bisphosphonates. It is therefore considered separately from the bisphosphonate trials to prevent dilution of any observed effect (Fig 2). There is evidence of significant heterogeneity between the bisphosphonate versus denosumab subgroups (I^2^ 86%, p = 0.007 test for subgroup differences) and important differences between the bisphosphonate trials and Smith 2012 [13] must be considered.

#### Overall survival

**Fig 3:**
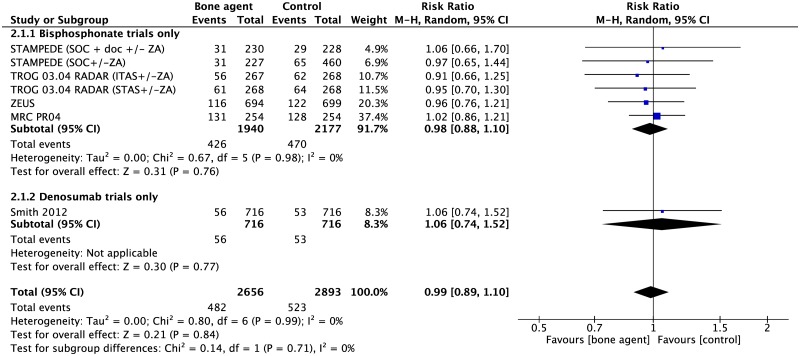
Forest plot for overall survival. Abbreviations: ITAS, intermediate term androgen suppression; STAS, short term androgen suppression; ZA, zoledronic acid.

There was no difference in all-cause mortality between osteoclast inhibitors and placebo/SOC (5 studies, 5549 participants, RR 0.99 95% CI 0.89–1.10, p = 0.84) (Fig 3).

#### Prostate cancer specific survival

**Fig 4:**
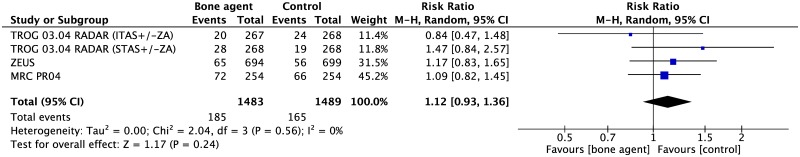
Forest plot for prostate cancer specific survival. Abbreviations: ITAS, intermediate term androgen suppression; STAS, short term androgen suppression; ZA, zoledronic acid.

There was no difference in prostate cancer mortality between bisphosphonates and placebo and/or SOC (3 studies, 2972 participants, RR 1.12 95%CI 0.93–1.36, p = 0.24) (Fig 4).

#### Mortality unrelated to prostate cancer

**Fig 5:**
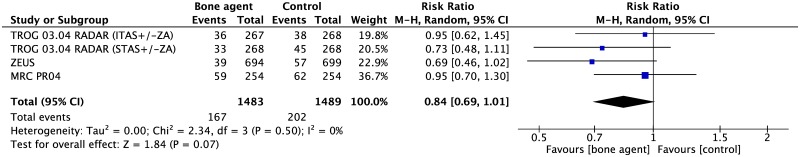
Forest plot for mortality unrelated to prostate cancer. Abbreviations: ITAS, intermediate term androgen suppression; STAS, short term androgen suppression; ZA, zoledronic acid.

There was a trend towards reduced non-prostate cancer related death with bisphosphonates (3 studies, 2972 participants, RR 0.84, 95%CI 0.69–1.01) (Fig 5). However, this did not attain significance (p = 0.07).

#### HRQOL and toxicity

Five studies [13, 19–22] reported on toxicity and one study [23] reported on HRQOL. Reporting measures differed between trials and only three trials [13, 20, 22] reported the use of standardized measurement common terminology criteria for adverse events (CTCAE) criteria. There was insufficient published data for a quantitative analysis, and qualitative results are summarized in Table 3. In general, there was a higher incidence of osteonecrosis of the jaw (5% or less) and hypocalcemia (2% or less) in the osteoclast inhibitor arms compared with placebo/SOC, although these rates were small.

**Table 3:** Toxicity and HRQOL outcomes.

Study	Reporting measure	Outcomes
**Smith 2012** [13]	CTCAEv3	Increased toxicity with denosumab; osteonecrosis of the jaw (33 vs. 0) and hypocalcemia (12 vs. 2)
**ZEUS** [21]	Unclear; no measure reported	Increased toxicity with ZA; influenza-like symptoms of general low severity such as muscle/bone pain, arthralgia, fever, nausea and dizziness; osteonecrosis of the jaw in 10 patients (9 vs. 1); hypocalcemia in five patients (4 vs. 1); femoral head osteonecrosis in one patient not considered ZA related (1 vs. 0).
**TROG 03.04 RADAR** [20, 23]	CTCAEv2; EORTC QLQC30 and PR25	The use of ZA for 18 months did not appear to be associated with any independent effects on patient reported outcomes and HRQOL. Nine participants had serious adverse events potentially related to ZA (osteonecrosis jaw (2), urticaria (1), syncope during infusion (1), hypotension after infusion (1), renal pain related to hydronephrosis (1), pyrexia and chest pain (1).
**MRC PR04** [19]	Non-standardized measure*	Increased toxicity with clodronate (gastrointestinal symptoms and increased lactate dehydrogenase levels)
**STAMPEDE** [22]	CTCAEv3.0	Thirty cases of osteonecrosis of the jaw in ZA arms of trial (10 in SOC + ZA; 20 in SOC + ZA + docetaxel)

Abbreviations: ZA, zoledronic acid; SOC, standard of care; CTCAE, common terminology criteria for adverse events; HRQOL, health related quality of life; EORTC, European Organisation for Research and Treatment of Cancer; QLQC30, quality of life questionnaire—core questionnaire; PR25, prostate cancer questionnaire

* adverse events defined as events leading to alteration in trial medication, hospitalization, prolongation of hospitalization or death.

## Discussion

This meta-analysis of four randomized controlled trials including 4,404 participants with high-risk, M0 prostate cancer did not show evidence of a significant difference in incidence of bone metastases between osteoclast inhibitors and placebo/SOC. However, caution is required in interpreting these results as pooling of the studies led to significant heterogeneity between the bisphosphonate and denosumab trials. Hence, the denosumab and bisphosphonate trials need to be considered separately.

Smith 2012 [13], is a randomized controlled trial of 1,432 participants that compared denosumab with placebo in the high-risk, M0, prostate cancer population and is the largest study included in our meta-analysis. It showed delay in onset of bone metastases when denosumab was compared with placebo (RR 0.83, p = 0.007) and is the only positive trial in the included studies. Further subgroup analysis, by Smith *et al* [24], demonstrated that shorter bone metastasis free survival was observed as PSA doubling time decreased below 8 months and that denosumab consistently increased bone metastasis free survival in participants with shorter PSA doubling times (median of 6, 7.2 and 7.5 months among men with PSA doubling times ≤10 (HR 0.84; p = 0.042), ≤6 (HR 0.77; p = 0.006), and ≤4 months respectively (HR 0.71; p = 0.004).

Denosumab has a fundamentally different mechanism of action compared to bisphosphonates. Denosumab is a fully human monoclonal IgG_2_ antibody which inhibits RANKL from binding to its receptor, RANK, a critical element for osteoclast formation, function and survival and thereby leads to loss of osteoclasts from bone surfaces [25]. It primarily acts in the extracellular milieu. In contrast, bisphosphonates, synthetic analogues of pyrophosphate, bind to bone mineral and need to be internalized to act upon cells. The exact mechanism is unknown, but they likely inhibit osteoclast function through intracellular effects, once taken up by mature osteoclasts at sites of bone resorption [25]. Moreover, osteoclast inhibitors appear to have a complex interaction with the bone marrow microenvironment, a rich source of bone-derived growth factors (e.g. transforming growth factor-beta, insulin-like growth factor) and immune cells, which are likely to play important roles in the regulation of tumor cell growth in bone [26–28]. In addition, denosumab and nitrogen-containing bisphosphonates (e.g. zoledronic acid) may also have osteoclast-independent, direct anti-tumor activity via different mechanisms of action, although further research is needed [29, 30]. Interestingly, there is preclinical evidence that suggests an immune-mediated anti-tumor effect mediated via RANKL inhibition [31, 32].

While the difference in drug mechanism of action is the most critical factor to explain the difference in efficacy between the bisphosphonate and denosumab trials, it is important to consider other differences in the trial designs and study populations that may have also contributed to heterogeneity. All participants in the denosumab trial [13] had castrate resistant disease and had to meet one of two PSA criteria (PSA ≥8 μg/L and/or PSA doubling time ≤10 months) making them higher risk candidates for bone metastases compared with the participants in the other studies. It was also the only trial that mandated regular (4 monthly) bone scans throughout the trial period. Given that the majority of bone metastases detected in Smith 2012 [13] were asymptomatic (440/605; 73%), the more regular imaging may have increased the sensitivity to detect a treatment effect. Furthermore, this was the only trial with central review of bone imaging for the entire trial cohort, reducing the risk of detection bias. Finally, it is the only study in the quantitative analysis that allowed concomitant antineoplastic therapy. However, the use of chemotherapy or biological agents was reported to be balanced between the intervention and comparator groups with no notable differences in treatment type.

When drug classes were considered separately, there was no evidence that bisphosphonates delay the onset of bone metastases (HR 1.20, p = 0.12, I^2^ 33%), in contrast to denosumab (RR 0.83, p = 0.007). Furthermore, in one of the bisphosphonate trials, the short term androgen suppression arm (STAS, 6 months) of the RADAR study [20], showed a higher incidence of new bone metastases in the bisphosphonate arm. It is unclear why a significant increase in bone progression was noted in STAS plus zoledronic acid compared with STAS alone, particularly as no difference was noted between the intermediate term androgen suppression arm of the same trial (18 months). In a post-hoc analysis, the difference between STAS plus zoledronic acid and STAS, was most notable in tumors with Gleason score less than 7, compared to a non-significant difference in tumors with Gleason score 8–10 [20]. We were unable to perform a subgroup analysis based on Gleason score due to insufficient published data and this deserves the attention of further research.

We did not demonstrate any impact of osteoclast inhibitors on either overall survival or prostate cancer specific survival. These results mirror those of a recently published review by Vale *et al* [33], despite the additional inclusion of the denosumab trial [13]. Interestingly, there was a non-significant trend towards decreased mortality unrelated to prostate cancer with bisphosphonates, consistent with recent work which showed a reduced all-cause mortality in the osteoporosis population independent of fracture prevention [34, 35]. However, it is important to view this result in the context of unaltered overall survival.

A quantitative analysis was unable to be performed on toxicity and HRQOL outcomes as the reporting methods differed between the trials. Most studies reported increased incidence of osteonecrosis of the jaw and hypocalcemia in the treatment arm but incidence rates were very low. It is important to note, however, that incidence rates were higher in the more recently performed trials likely due to more diligent detection protocols. In addition, Smith 2012 [13] reported that 94% of those men who had osteonecrosis of the jaw had pre-existing oral risk factors, including tooth extraction, poor oral hygiene and dental application use. It is reasonable to assume that if men were selected more carefully for osteoclast inhibitor therapy the incidence of osteonecrosis of the jaw could be reduced. Similarly, in terms of the complication of hypocalcemia, no trial reported participant’s 25-hydroxyvitamin D levels prior to commencing treatment and only two trials [16, 21] mandated concomitant vitamin D3 and calcium supplementation. With more careful screening of participants and supplemental calcium and vitamin D3, the incidence of hypocalcemia with osteoclast inhibitors could also be reduced.

There are several limitations to our review. Firstly, frequency of drug administration differed between the zoledronic acid studies (monthly vs. q3monthly). Exploratory studies suggest that cancer patients with elevated bone turnover markers have a higher risk of bone progression [36] and that q3monthly administration of zoledronic acid is not adequate to suppress markers of bone turnover [37, 38]. In contrast to the denosumab trial [13], many of the bisphosphonate trials included in our quantitative analysis did not measure markers of bone turnover to confirm adequate suppression of bone resorption. Secondly, there was large variation in characteristics of the participants in terms of their risk of bone metastases and more importantly, their prognosis (e.g. hormone status, PSA, Gleason score, tumor stage). Thirdly, the majority of the trials did not mandate a regular imaging schedule but instead allowed imaging at the clinician’s discretion potentially resulting in under-detection of bone metastases. Finally, in the absence of bone marrow biopsy [39] and imaging with high sensitivity for occult bone metastases (e.g. ^68^Ga-PSMA PET/CT or whole body diffusion weighted MRI [40–42]), it is likely that many patients, especially those with castrate resistant disease, had the presence of micrometastases or small metastases below the detection of scintigraphy, on trial enrollment. Hence, our analysis was only able to assess the impact of osteoclast inhibitor therapy on the delay of onset of radiologically-detectable bone metastases, rather than their true prevention.

Overall, our meta-analysis demonstrates limited impact of osteoclast inhibitors in men with high-risk, M0 prostate cancer however interpretation is limited by significant heterogeneity between the bisphosphonate and denosumab studies likely due to pooling of drug classes with different mechanisms of action. While there was no benefit seen with bisphosphonates, the denosumab trial, Smith 2012 [13], demonstrated a significant delay in the onset of bone metastases in this population. Our results highlight the critical need for further trials selecting men at true high risk of bone metastases, which incorporate castrate resistant disease status, short PSA doubling times and high Gleason score at baseline. Furthermore, as modern imaging with greater sensitivity to detect occult bone metastases, like ^68^Ga-PSMA PET/CT, becomes incorporated into practice, it will become increasingly difficult to apply evidence from these trials in the bygone era.

## Supporting information

S1 PRISMA Checklist (DOC)

S1 Appendix Search strategy. (DOCX)

S1 Table Study characteristics. (DOCX)

S2 Table Risk of bias tables. (DOCX)

S1 Fig Risk of bias summary for included trials. (EPS)

S2 Fig Risk of bias graph for included trials. (EPS)

S3 Fig Forest plot for incidence of new bone metastases in bisphosphonate only trials with RADAR STAS arm excluded. Abbreviations: ITAS, intermediate term androgen suppression; STAS, short term androgen suppression; ZA, zoledronic acid.
(EPS)
