# Supplementary material for: Osteoclast inhibitors to prevent bone metastases in men with high-risk, non-metastatic prostate cancer: A systematic review and meta-analysis
Source: PLoS One. 2018 Jan 25;13(1):e0191455. doi: 10.1371/journal.pone.0191455 (PMC5784941; doi:10.1371/journal.pone.0191455)
Supplement: S1 Appendix — (DOCX) [file pone.0191455.s002.docx]

**S1 Appendix. Search strategy.**

The search strategy used the following terms:

‘prostatic neoplasms’ OR ‘prostat$ adj3 (cancer$ or carcinoma$ or malignan$ or tumo?r$ or neoplas$ or adeno$).mp’; ‘diphosphonates’ OR ‘denosumab’ OR ‘(bisphosphonate$ or diphosphonat$).af OR ‘alendron$.af’ OR ‘fosamax.af’ OR ‘clodron$.af’ OR ‘bonefos.af’ OR ‘CL2MDP.af’ OR ‘loron.af OR ‘ostac.af’ OR ‘etidron$.af’ OR ‘didrocal.af’ OR ‘didronel.af’ OR ‘EHDP.af’ OR ‘ibandron$.af’ OR ‘bondranat.af’ OR ‘incadron$.af’ OR ‘YM175.af’ OR ‘YM175.af’ OR ‘minodron$.af’ OR ‘YM529.af’ OR ‘YM529.af’ OR ‘neridron$.af’ OR ‘AHDP.af’ OR ‘olpadron$.af’ OR ‘OPD.af’ OR ‘pamidron$.af’ OR ‘APD.af’ OR ‘aredia.af’ ‘pamisol.af; OR ‘risedron$.af’ OR ‘actonel.af’ OR ‘tiludron$.af’ OR ‘skelid.af’ OR ‘zometa.af’ OR ‘zoledron$.af’ OR ‘denosum$.af’ OR ‘prolia.af’; randomized controlled trial.pt’ OR ‘controlled clinical trial.pt’ OR ‘randmized.ab’ OR ‘placebo.ab’ OR ‘clinical trials as topic.sh’ OR ‘randomly.ab’ OR ‘trial.ti’ OR ‘exp animals/ not humans.sh’.
