# Supplementary material for: Osteoclast inhibitors to prevent bone metastases in men with high-risk, non-metastatic prostate cancer: A systematic review and meta-analysis
Source: PLoS One. 2018 Jan 25;13(1):e0191455. doi: 10.1371/journal.pone.0191455 (PMC5784941; doi:10.1371/journal.pone.0191455)
Supplement: S1 Table — (DOCX) [file pone.0191455.s003.docx]

**S1 Table. Study characteristics.**

**Zometa 704**

| **Methods** | Randomised double blind placebo controlled trial. Accrual period: September 1999 – September 2002. n=398 (target accrual 991). In December 2001, the Data and Safety Monitoring Board placed the study on hold due to low observed event rates. In September 2002, the study was terminated. Due to the low event rate and early termination, efficacy was unable to be evaluated. Data from the placebo group only was used for a subsequent analysis to identify men at high risk of developing bone metastases.  **Evaluation of bone metastases:** Bone scans were performed every 4 months. No report of central review. Additional bone scans were performed to evaluate new symptoms suggestive of bone metastases but not based on PSA rises. New bone scan metastases were confirmed with plain radiographs, magnetic resonance imaging or computed tomography. |
| --- | --- |
| **Participants** | **Inclusion criteria:** Prostate cancer patients with no radiographic evidence of metastases and PSA progression despite ADT. PSA progression was defined as three consecutive rises in serum PSA (measured at least 2 weeks apart), initial PSA rise within 10 months of study entry, and last PSA ≥ 150% of nadir value.  **Patient characteristics:** Mean age 73 years, 34% prior radical prostatectomy, 17% N1 disease at diagnosis, mean serum PSA at study entry 35.4 ng/ml, 29% Gleason score ≥8 at diagnosis, performance status ECOG 0-1 100%. |
| **Interventions** | **Control:** placebo  **Treatment:** zoledronic acid (4 mg intravenously every 4 weeks) for 49 treatments  All patients prescribed supplemental calcium 500 mg/day and vitamin D 400-500 U/day |
| **Outcomes** | **Primary endpoint**: Time to first bone metastasis  **Secondary endpoints:** overall survival and bone metastasis- free survival (time to first positive bone scan/radiograph).  **Follow up:** Patients were evaluated monthly for 48 months. Symptom assessment and physical examination performed at each visit. Serum PSA was measured at baseline and then every 4 months. |
| **Notes** | Treatment with secondary hormonal therapy or chemotherapy was allowed at the discretion of the treating physician |

**ZEUS**

| **Methods** | Open label multinational, multicentre randomised controlled trial in 13 European centres. Accrual period: June 2004 – August 2007. n=1433. Median follow up 4.8 years.  Intention to treat analysis provided.  **Evaluation of bone metastases:** assessed by local bone imaging procedures, mainly bone scans, in cases with pain or PSA ≥ 10 ng/ml. After 4 years, bone imaging procedures were recommended. Additional imaging was recommended to confirm bone scan hotspots however only 53% of patients with a positive bone scan had confirmative imaging. For a more accurate understanding of the number of bone metastases the protocol was amended so that bone images could be retrospectively collected for independent and blinded central review. |
| --- | --- |
| **Participants** | **Inclusion criteria:** M0 prostate adenocarcinoma with or without prior local curative prostatectomy or radiotherapy (no more than 6 mo between curative  treatment and baseline) and a Karnofsky performance status ≥90. At  least one of three high-risk factors had to be present: Gleason score 8–10,  node-positive disease, or prostate-specific antigen (PSA) at diagnosis ≥20 ng/ml.  **Patient characteristics:** Mean age 68 years, 44.7% prior local treatment, 55.1% PSA ≥ 20 ng/ml at diagnosis, 62.2% Gleason score ≥8 at diagnosis, 23.9% N1 disease at baseline, performance status ECOG 0-1 100%, white race 94%. |
| **Interventions** | **Control:** standard ADT only, if applicable  **Treatment:** standard ADT, if applicable, plus zoledronic acid 4mg intravenously every 3 months for up to 4 years  All patients received concomitant calcium 500mg and vitamin D 400-500 IU daily |
| **Outcomes** | **Primary endpoint**: Proportion of patients with bone metastases at 4 ± 0.5 yr  **Secondary endpoints:** adverse events, overall survival, and time to bone metastases.  Follow up protocol not provided. |
| **Notes** | Patients receiving ADT at study entry could later stop ADT and patients not receiving ADT could later start ADT. Prior treatment with bisphosphonates, chemotherapy for prostate cancer or ADT monotherapy excluded. |

**MRC PR04**

| **Methods** | Randomised double-blind placebo controlled phase III trial. Multicentred – 26 UK sites and 1 NZ site. Accrual period: June 1994 – December 1997. n= 508. Median follow up (survival) 11.5 years.  Intention to treat analysis provided.  **Evaluation of bone metastases:** No information provided on how symptomatic bone metastases were confirmed radiologically. |
| --- | --- |
| **Participants** | **Inclusion criteria:** Diagnosis of locally advanced prostate adenocarcinoma (T2-T4, N0, N+ or NX, M0) within 36 months before study entry  **Patient characteristics:** Median age 69 years, 47% had T3/4 disease, 81% were known to be node negative, 96% WHO performance status 0-1. Primary treatment: 42% radiotherapy only, 28% radiotherapy plus ADT, 24% ADT only. The proportion receiving hormone therapy was greater in patients with worse tumour stage. |
| **Interventions** | Randomised 1:1  **Control:** local standard of care plus placebo (four tablets identical to sodium clodronate)  **Treatment:** local standard of care plus daily oral sodium clodronate 2080 mg/day (four 520mg tablets) for a maximum of 5 years.  **Compliance**: Median time on trial medication was 47 months for sodium clodronate and 60 months for placebo. |
| **Outcomes** | **Primary endpoint**: Symptomatic bone-metastasis free survival, defined as time from random assignment to development of symptomatic bone metastases or death from prostate cancer.  **Secondary endpoints:** Overall survival, toxicity, rate of events affecting bone during the trial and type of progressive disease (bone versus non-bone).  **Follow up:** Six weeks from randomisation, then every 6 months for 2 years and then yearly until death. Clinical examination plus FBC, UEC, biochemistry, LFT and assessment if pain requiring analgesia was present. There were no routine scans for asymptomatic bone metastases. |
| **Notes** | Prior treatment with bisphosphonates or long term hormone therapy excluded |

**Smith 2012**

| **Methods** | Randomised double-blind placebo controlled phase III trial. Multicentred – 319 centres from 30 countries. Accrual period: Feb 2006 – July 2008. n= 1432. Median follow up not reported.  Intention to treat analysis provided.  **Evaluation of bone metastases:** All patients had a radioisotope bone scan during screening with subsequent CT, MRI or plain radiograph if needed to exclude bone metastases. Patients with imaging results that were equivocal or consistent with bone metastases were excluded. Bone scans were done every 4 months to detect bone metastases and a confirmed diagnosis of bone metastases required a second imaging modality (CT, MRI, or plain radiograph). |
| --- | --- |
| **Participants** | **Inclusion criteria:** Histologically confirmed castrate resistant prostate cancer (total serum testosterone < 1.72 nmol/L, 50 ng/dL with three consecutive increasing PSA tests separated by at least 2 weeks). On ADT for at least 6 months and at high risk for bone metastases (PSA 8.0 ug/L or higher within 3 months before randomisation and/or PSA doubling time of 10 months or less). M0 disease.  **Patient characteristics:** Median age 74 years, median PSA 12.5 ug/L, 48% had PSA 8.0 ug/L or higher within 3 months of randomisation and PSA doubling time 10 months or less, 13% had lymphatic disease, 32% had gleason score 8-10 at diagnosis, 45% had local therapy with prostatectomy or radiotherapy or both. 99.7% WHO performance status 0-1. 84-85% white race. |
| **Interventions** | Randomised 1:1  **Control:** local standard of care plus subcutaneous placebo every 4 weeks  **Treatment:** local standard of care plus subcutaneous denosumab 120mg every 4 weeks  **Compliance**: Median time on trial medication was 19 months for denosumab and 18 months for placebo. |
| **Outcomes** | **Primary endpoint**: Bone-metastasis-free survival, as determined by time to first occurrence of bone metastasis (symptomatic or asymptomatic) or death from any cause.  **Secondary endpoints:** Time to first bone metastasis (symptomatic or asymptomatic, excluding deaths) and overall survival (including deaths on study and during follow-up).  **Tertiary endpoints:** Overall prostate cancer progression, prostate cancer progression-free survival, proportion of patients with symptomatic bone metastasis and change from baseline in PSA concentration. Changes from baseline in bone turnover markers were also assessed. Safety was assessed at regular intervals and  included adverse events graded by Common Terminology Criteria for Adverse Events (CTCAE) version 3.  **Follow up:** Radioisotope bone scan every 4 months, oral examinations every 6 months and radiographic skeletal surveys once yearly. Study procedures also included medical history and physical examination, vital signs, haematological tests, serum chemistry, denosumab concentration and antidenosumab antibody assays, urine collection, central laboratory PSA measurement, and testosterone assessments. |
| **Notes** | Prior treatment with denosumab or intravenous bisphosphonates excluded. Prior oral bisphophosphonates allowed if <3 years continuous use and washout period of ≥1 year before randomisation. Antineoplastic and concomitant treatments deemed necessary were allowed before enrolment and on study. |

**TROG 03.04 RADAR**

| **Methods** | Randomised, open-label, phase III trial with a 2 x 2 factorial design. Multicentred – 23 centres across Australia and New Zealand. Accrual period: Oct 2003 – Aug 2007. n= 1071. Median follow up 7.4 years.  Intention to treat analysis provided.  **Evaluation of bone metastases:**  Distant progression diagnosed by bone scintigraphy, plain radiology or CT. Frequency of radiological examinations not specified nor what clinical parameters/symptoms prompted a radiological examination. No report of central review. |
| --- | --- |
| **Participants** | **Inclusion criteria:** Histologically confirmed prostate adenocarcinoma without lymph node or systemic metastases, stage (T2b-4 primary tumour) with any Gleason score and a baseline PSA or stage T2a primary tumour with Gleason score 7 or higher and a baseline PSA of at least 10 ug/L  **Patient characteristics:** Median age 68.8 years, 35% Gleason score > 7, 37% had T3/4 disease, 32% PSA >20 ug/L, 80% high risk by D’Amico risk group classification, ECOG 0-1 100%. |
| **Interventions** | Randomised 1:1:1:1  **Control group:** Short-term (6 months) or intermediate-term (18 months) androgen suppression (leuprorelin IMI 22.5mg every 3 months) and radiotherapy alone;  **Treatment groups:** Leuprorelin (short or intermediate-term) plus 18 months months of zoledronic acid (4mg every 3 months intravenously) |
| **Outcomes** | **Primary endpoint**: PSA relapse-free survival (at beginning of trial in 2003). Changed to prostate cancer specific mortality (Dec 2011) after findings of TROG 96.01 showed that PSA progression as an event was not strongly prognostic for metastases and death.  **Secondary endpoints:** PSA progression, local progression, distant progression, bone progression, time to secondary therapeutic intervention, all-cause mortality.  **Follow up:** Every 3 months for up to 30 months then every 6 months for up to 5 years after randomisation, then yearly for a further 5 years. At every clinic visit, PSA and outcomes reported by clinician and a digital rectal exam was performed. |
| **Notes** | Lymph node metastases excluded. Previous ADT and prostatectomy excluded. Current bisphosphonate treatment excluded – no washout period specified for recently ceased bisphosphonates. |

**STAMPEDE**

| **Methods** | Randomised controlled trial using a multiarm, multistage platform design. Accrual period: Oct 2005 – Mar 2013. n= 2962. Median follow up 3.6 years.  Intention to treat analysis provided.  **Evaluation of bone metastases:**  Bone progression assessed as part of failure-free survival (intermediate primary end point). No report of how often radiological examinations were undertaken or what clinical parameters/symptoms prompted a radiological examination. No report of central review. |
| --- | --- |
| **Participants** | **Inclusion criteria:**  Newly diagnosed prostate cancer that was metastatic, node positive or high risk locally advanced (with at least two of T3/4, Gleason score of 8-10 and prostate specific antigen ≥40 ng/mL); or previously treated with radical surgery, radiotherapy, or both and relapsing with high risk features. All patients intended for long term ADT started no longer than 12 weeks before randomisation.  **Patient characteristics:** Median age 65 years, median PSA 65ng/ml, 94% newly diagnosed, 71% Gleason score >7. |
| **Interventions** | Randomised 2:1:1:1 to SOC only, SOC plus ZA, SOC plus docetaxel, SOC plus docetaxel and ZA.  **Control group:** SOC. SOC was ADT for at least 2 years with gonadotropin-releasing hormone agonists or antagonists or, oral anti-androgens alone in patients with non-metastatic disease between 2006 and 2011. Orchidectomy allowable as alternative to drug therapy.  **Treatment groups:**  SOC plus ZA (4mg every 3 weeks for 6 cycles, then 4 weekly until 2 years)  SOC plus docetaxel (75mg/m2 3 weekly for 6 cycles with prednisone 10mg daily).  SOC plus docetaxel and ZA (4mg every 3 weeks for 6 cycles, then 4 weekly until 2 years) |
| **Outcomes** | **Primary endpoint**: Overall survival (definitive) and failure-free survival (intermediate) defined as biochemical failure, local or distant progression or death from prostate cancer.  **Follow up:** 6 weekly to 6 months, 12 weekly to 2 years, 6 monthly to 5 years, then annually. PSA was measured at every follow up, further tests were at the clinician’s discretion. |
| **Notes** | Radiotherapy at 6-9 months after randomisation was encouraged for participants with N0M0 disease until Nov 2011, then mandated. Radiotherapy was optional for participants with N+M0 disease. |
