# Supplementary material for: Osteoclast inhibitors to prevent bone metastases in men with high-risk, non-metastatic prostate cancer: A systematic review and meta-analysis
Source: PLoS One. 2018 Jan 25;13(1):e0191455. doi: 10.1371/journal.pone.0191455 (PMC5784941; doi:10.1371/journal.pone.0191455)
Supplement: S2 Table — (DOCX) [file pone.0191455.s004.docx]

**S2 Table. Risk of bias tables.**

**Zometa 704**

| **Bias** | **Support for judgement** |
| --- | --- |
| **Random sequence generation (selection bias)** | **Unclear risk**: Randomised method not given. |
| **Allocation concealment (selection bias)** | **Unclear Risk:** No information on allocation concealment |
| **Blinding of participants and personnel (performance bias)** | **Low Risk:** Double blinded, placebo controlled |
| **Blinding of outcome assessment (detection bias)** | **Unclear risk:** Central review of imaging not mentioned. |
| **Incomplete outcome data (attrition bias)** | **Unclear Risk:** No data provided on attrition. |
| **Selective reporting (reporting bias)** | **Unclear Risk:** Endpoints reported for control arm only. |
| **Overall bias** | Unclear |

**ZEUS**

| **Bias** | **Support for judgement** |
| --- | --- |
| **Random sequence generation (selection bias)** | **Low risk**: Randomised with minimisation method described by Pocock (REF) over three stratification factors. Baseline characteristics similar. |
| **Allocation concealment (selection bias)** | **Unclear Risk:** No information on allocation concealment |
| **Blinding of participants and personnel (performance bias)** | **High Risk:** Open label. No placebo. Patients on ZA had more frequent visits to the clinic which could lead to more frequent bone imaging and more patients diagnosed with bone metastases. However, the bone imaging procedures were comparable in both arms. |
| **Blinding of outcome assessment (detection bias)** | **Low risk:** Central review of bone images performed after clinical blinding (available only on a subset of patients). |
| **Incomplete outcome data (attrition bias)** | **Low Risk:** ITT analysis. 22 and 18 patients excluded from ZA and control group respectively. Exclusions balanced by group. |
| **Selective reporting (reporting bias)** | **Low Risk:** All endpoints reported. |
| **Overall bias** | Low |

**MRC PR04**

| **Bias** | **Support for judgement** |
| --- | --- |
| **Random sequence generation (selection bias)** | **Low risk**: Randomised with minimisation method over five stratification factors. Baseline characteristics similar. |
| **Allocation concealment (selection bias)** | **Low Risk:** Random assignment was undertaken centrally. |
| **Blinding of participants and personnel (performance bias)** | **Low Risk:** Participants and personnel blinded. All patients allocated unique trial number and unique drug number. |
| **Blinding of outcome assessment (detection bias)** | **Low Risk:** Personnel blinded to treatment allocation when performing clinical assessment and review of cause of death. Unclear if radiological assessment was blinded and centralised. |
| **Incomplete outcome data (attrition bias)** | **Low risk:** First analysis: ITT analysis. No randomised patients excluded. Second analysis with longer follow-up: 34 patients excluded because not registered in England or Wales. Three patients excluded because not flagged with UK national health service information centre. |
| **Selective reporting (reporting bias)** | **Low Risk:** All endpoints reported. |
| **Overall bias** | Low |

**Smith 2012**

| **Bias** | **Support for judgement** |
| --- | --- |
| **Random sequence generation (selection bias)** | **Low risk**: Computer generated randomisation schedule prepared by individual independent of the study team. Two stratification factors. Randomly permuted block design with a block size of four applied. |
| **Allocation concealment (selection bias)** | **Low Risk:** Masked allocation to treatment via interactive voice response system. |
| **Blinding of participants and personnel (performance bias)** | **Low Risk:** All patients, investigators and people involved in study conduct were blinded to treatment allocation |
| **Blinding of outcome assessment (detection bias)** | **Low Risk:** All radiographic assessments performed by a central reader in a masked fashion with double-reader confirmation and adjudication by a third reader in case of disagreement. |
| **Incomplete outcome data (attrition bias)** | **Low Risk:** ITT analysis. Three patients excluded after randomisation – review activities and oversight of institutional review board not ensured. |
| **Selective reporting (reporting bias)** | **Low Risk:** Primary and secondary endpoints assessed hierarchically. The  secondary endpoint of time to first bone metastasis was tested only if the primary endpoint of bone-metastasis free survival was significant in favour of denosumab. If time to first bone metastasis was also significant in favour of denosumab, overall survival was tested. Nevertheless, all end points were reported so therefore low risk. |
| **Overall bias** | Low |

**TROG 03.04 RADAR**

| **Bias** | **Support for judgement** |
| --- | --- |
| **Random sequence generation (selection bias)** | **Low risk**: Computer generated minimisation. Five stratification factors |
| **Allocation concealment (selection bias)** | **Low Risk:** Central trials office (computer based) |
| **Blinding of participants and personnel (performance bias)** | **High Risk:** Open label. No placebo. Treatment was not masked to investigators, patients, or the study statistician. However, RADAR trial endpoints committee were blinded to treatment group. |
| **Blinding of outcome assessment (detection bias)** | **Unclear Risk:** RADAR trial endpoints committee were blinded to treatment group. No mention of centralised radiological review for assessment of local, distant or bone progression. |
| **Incomplete outcome data (attrition bias)** | **Low Risk:** ITT analysis. All randomised patients accounted for. |
| **Selective reporting (reporting bias)** | **Low Risk:** All end points reported |
| **Overall bias** | Low |

**STAMPEDE**

| **Bias** | **Support for judgement** |
| --- | --- |
| **Random sequence generation (selection bias)** | **Low risk**: Minimisation with random element of 80%. Eight stratification factors. |
| **Allocation concealment (selection bias)** | **Low Risk:** Central randomisation using computerised algorithm |
| **Blinding of participants and personnel (performance bias)** | **Unclear Risk:** Open label. No placebo. |
| **Blinding of outcome assessment (detection bias)** | **Low Risk:** Cause of death determined by masked central review. Frequency of radiological examinations and prompts for radiological examination for local or distant progression assessment not specified. Central radiological review not specified. |
| **Incomplete outcome data (attrition bias)** | **Low Risk:** ITT analysis. All randomised patients accounted for. |
| **Selective reporting (reporting bias)** | **Low Risk:** All end points reported |
| **Overall bias** | Low - possible detection bias from lack of central radiological review which may affect outcome measures of disease progression but not OS. Only OS used in our quantitative analysis. |
